# Physicochemical Parameters and Multivariate Analysis to Predict the Sensory Quality in Specialty Coffee from Panama

**DOI:** 10.1021/acsomega.4c10914

**Published:** 2025-03-28

**Authors:** Aracelly Vega, Stephany M. Reyes, Jose Troestch

**Affiliations:** † Centro de Investigación en Recursos Naturales, Facultad de Ciencias Naturales y Exactas, Universidad Autónoma de Chiriquí, 0427 Chiriquí, Panamá; ‡ Sistema Nacional de Investigación de Panamá (SNI), Secretaría Nacional de Ciencia, Tecnologías e Innovación, 0816 Panamá, Panamá; § Laboratorio de Bromatología, Estación Experimental de Gualaca, Instituto de Innovación Agropecuaria de Panamá (IDIAP), 0436 Chiriquí, Panamá

## Abstract

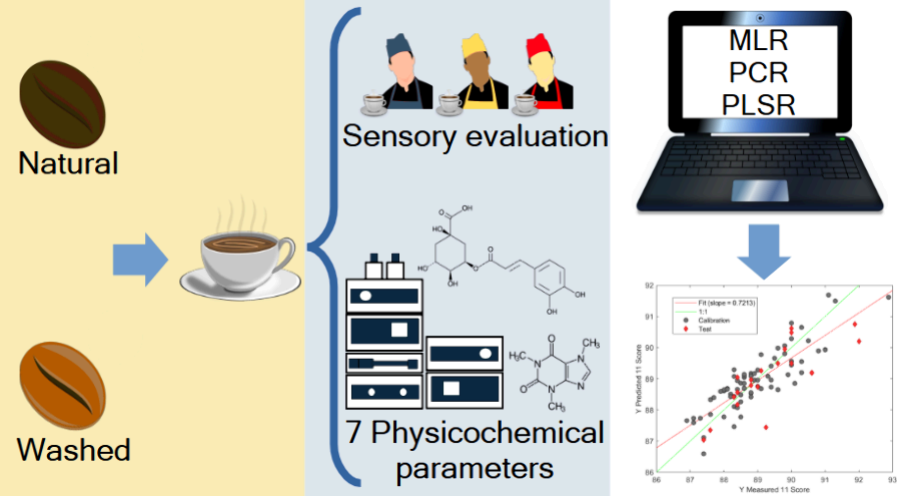

This study assessed the effectiveness of various multivariate calibration models in predicting the sensory evaluation scores of specialty coffee produced in Panama. The predictions were based on seven key physicochemical parameters of the beverage, considering the processing method used (natural or washed). To construct the models, three algorithms, Multiple Linear Regression (MLR), Principal Component Regression (PCR), and Partial Least Squares Regression (PLSR), were employed, analyzing data sets for natural, washed, and combined processing methods. Model quality was evaluated using metrics such as the coefficient of determination (R^2^), root-mean-square error (RMSE) for cross-validation and prediction, and the residual predictive deviation (RPD). Among the physicochemical parameters, titratable acidity, soluble solids, and protein content showed a positive correlation with sensory scores, whereas pH exhibited an inverse relationship. The best-performing MLR and PCR models were those for the natural process, achieving R^2^p, RMSEp, and RPD values of 0.8293, 0.4239, and 2.34 for MLR and 0.7233, 0.5322, and 1.86 for PCR, respectively. Across all algorithms, models built exclusively with data from a single processing method consistently outperformed those that combined samples from both processes. PLSR models further demonstrated this trend, with R^2^p values of 0.7639 and 0.8306, RMSEp of 0.6891 and 0.3948, and RPD values of 2.07 and 2.51 for the washed and natural processes, respectively. In conclusion, the study highlights the critical importance of considering processing methods when developing multivariate models to predict the sensory evaluation scores of specialty coffee. Models built with samples from a uniform processing method yielded significantly better performance than those developed using mixed-process data sets.

## Introduction

Since the 1990s, the term “specialty coffee” has been synonymous with the highest quality green coffee beans, characterized by exceptional and distinctive flavors.^1^ A specialty coffee is obtained through a standardized production process designed to ensure quality and accentuate the beverage’s unique characteristics.^2^ In recent years, global demand for specialty coffee has surged, with such coffees being defined by the Specialty Coffee Association (SCA) as those scoring above 80 points in a cupping test.^3^

Coffee quality is influenced by a range of factors, including soil and climate conditions, genetic variety, harvest and postharvest processes, roasting, and storage.^4^ These elements either enhance or diminish the chemical compounds responsible for coffee’s sensory attributes. Among these factors, the postharvest stage holds particular significance, as it can adjust and refine the bean’s intrinsic qualities, thereby affecting its body, acidity, and sweetness.^5^ During postharvest processing, two predominant methods are widely utilized: the natural (dry) method and the washed (wet) method. The natural method, the oldest and least technically demanding, involves drying ripe coffee cherries under the sun for 12 to 15 days until they achieve a moisture content of 10–12%. Afterward, a machine removes the dried outer layers, including the pulp, mucilage, and parchment.^6^ This method produces coffee with slightly acidic, sweet, mild, and bitter flavor profiles. Conversely, the washed process entails more intricate steps: the mechanical removal of the coffee skin and pulp, microbial fermentation to degrade the mucilage, and subsequent sun drying.^7^ This method enhances the development of aromatic compounds and increases acid concentrations, yielding a distinct flavor profile compared to the natural process.^8^

The sensory evaluation of coffee quality is conducted via the cupping test, an international standard recommended by the SCA. Certified cuppers assess attributes such as fragrance, aroma, flavor, acidity, astringency, bitterness, body, aftertaste, balance, cleanliness, and overall impression. Each attribute is scored individually, and the sum represents the coffee’s overall quality.^4,9^ However, the cupping test has been criticized for its subjectivity, limited availability of certified cuppers, inconsistencies in reproducibility, and sensory perception variability among evaluators. Additionally, it is time-consuming and sometimes leads to inaccurate conclusions.^9^

To address these challenges, recent research has focused on predicting coffee sensory quality using chemical compositions of roasted beans combined with analytical techniques and multivariate analysis.^10−13^ These approaches employ methods such as partial least-squares regression (PLSR), principal component regression (PCR), and multiple linear regression (MLR). Additionally, the use of machine learning algorithms such as random forest (RF), gradient boosting machine (GBM), and artificial neural networks (ANN), among others, allows capturing potential nonlinear relationships between variables, leading to more accurate and robust models. These methods are particularly useful when working with large data sets.^14^ Multivariate techniques are increasingly applied in food quality research, offering alternatives to traditional statistical methods for developing predictive calibration models.^15^

MLR predicts a dependent variable based on multiple independent predictors, establishing a linear relationship between them.^16^ Due to its simplicity, it has been considered a fundamental tool in social and natural sciences. However, its application requires considering the sample size and the correlation of the predictor variables.^17^ PCR addresses multicollinearity issues by combining principal component analysis (PCA) with MLR,^18^ reducing the dimensionality of the original data while preserving the maximum variance.^19^ PLSR, similar to PCA in its mathematical foundation, projects high-dimensional data onto linear subspaces but utilizes supervised learning, unlike PCA’s unsupervised approach.^20^ It is a widely used technique when the number of observations is smaller than the number of features.^21^ These techniques have been applied to predict sensory quality in various foods, including wine, rice, fruits, cheese, and coffee. However, no comparative study has evaluated the performance of these three algorithms in predicting the sensory quality of specialty coffee based on its physicochemical parameters.^22−25^ Therefore, the objective of this study was to assess the potential of PLSR, PCR, and MLR regression models in predicting the sensory scores of specialty coffee produced in Panama, considering the physicochemical characteristics of the beverage and the processing method employed (natural or washed).

## Materials and Methods

### Coffee Samples

A total of 96 samples of *Coffea arabica* were obtained from the Specialty Coffee Association of Panama (SCAP). These samples originated from the districts of Boquete, Renacimiento, and Tierras Altas and were part of the “Best of Panama” tasting events held in 2018 and 2019.

### Sensory Evaluation

Prior to sensory analysis, the samples were categorized into batches based on their year and processing methods (natural or washed). For each sensory evaluation, 13 g of ground coffee were brewed with 140 g of water heated to 96 °C. The evaluation followed the SCA protocol, involving a panel of 16 certified cuppers. Fragrance was assessed within 15 min of grinding by removing the lid and smelling the dry grounds. Aroma was evaluated 3–5 min after brewing by breaking the crust (stirring three times) and sniffing the foam that runs off the back of the spoon. Flavor and Aftertaste were evaluated 8–10 min postinfusion when the beverage cooled to approximately 71 °C. The coffee was aspirated to cover as much of the palate as possible. The acidity, body, and balance were evaluated as the beverage cooled further, between 71 and 60 °C. Sweetness, Uniformity, and Cleanliness were Rated as the coffee neared room temperature (below 37 °C). The overall score evaluation was conducted at 21 °C, where the panel assigned taster points by combining scores from all sensory attributes.^26^ The final sensory evaluation is the average of the scores given by the 16 cuppers.

For physicochemical analysis, three cups were randomly selected from each batch and combined to create a composite sample (∼100 mL). These samples were stored in amber glass bottles at 8–15 °C until further analysis.

### Physicochemical Parameters

pH was determined using an OAKTON pH 1100 series potentiometer. Brix was measured using the AOAC 932.14C method with an AGON 0–32% refractometer (Thermo Fisher Scientific). Density was measured via the gravimetric method. Titratable Acidity was determined by titrating 10 mL of coffee beverage diluted with 100 mL of distilled water, with phenolphthalein used as an indicator, and the solution titrated with 0.1 M NaOH until a color change occurred. Protein Content was measured using the Kjeldahl method (AOAC 2001.11), applying a conversion factor of 6.25 to calculate the percentage of protein.^27^

The content of caffeine and 5-caffeoylquinic acid (5-CQA) was analyzed using an Agilent 1260 Infinity system equipped with diode array detection (HPLC-DAD). Separation was achieved using a ZORBAX SB-C18 column (4.6 mm × 150 mm, 5 μm). The mobile phases consisted of water with 0.02% trifluoroacetic acid (A) and methanol with 0.02% trifluoroacetic acid (B). The analysis employed a 20 μL injection volume, a flow rate of 1.0 mL/min, a column temperature of 25 °C, and detection wavelengths of 325 nm for chlorogenic acids and 280 nm for caffeine. The gradient method proposed by Vega et al. was followed.^28^ All measurements of the seven physicochemical parameters analyzed, including sample preparation, were performed in triplicate.

### Statistical Analysis

The statistical analysis of the chemical variables, calibration, and prediction partitioning, correlation analysis, and principal component analysis (PCA) was performed using R^29^ and RStudio.^30^ The following packages were employed: stats,^29^ caret,^31^ corrplot,^32^ car,^33^ ggplot2,^34^ FactoMineR,^35^ factoextra,^36^ and dplyr.^37^ Model development was conducted using SOLO software version 9.2 (Eigenvector Research, Inc.).

To predict the sensory evaluation scores assigned by the cupping panel based on physicochemical parameters (Brix, pH, density, titratable acidity, protein content, caffeine, and 5-CQA), multivariate calibration models were developed using multiple linear regression (MLR), principal component regression (PCR), and partial least-squares regression (PLSR). The data set comprised 55 washed coffee samples and 41 natural coffee samples, which were randomly split into calibration (80%) and prediction (20%) sets. Separate regression models were developed for each processing method (natural and washed), along with models that combined all samples regardless of processing type.

Cross-validation was performed using the Venetian blinds method (with 10 splits and a blind thickness of 1) to optimize the regression models. Model performance was assessed using several metrics: Root Mean Square Error of Calibration (RMSEc), Root Mean Square Error of Prediction (RMSEp), Coefficient of Determination for Cross-validation (R^2^cv), Coefficient of Determination for Prediction (R^2^p), Root Mean Square Error of Cross-Validation (RMSEcv) and the residual predictive deviation (RPD). The RMSEcv values were derived from the predicted scores of the calibration samples, and the lowest RMSEcv value was used to determine the optimal parameters for each regression model. A paired Student’s *t*-test was applied to evaluate whether statistically significant differences existed between the sensory scores assigned by the cupping panel and those predicted by the selected models (*p* < 0.05). This ensured that the predictive models were not only accurate but also aligned with the sensory panel’s evaluations.

## Results and Discussion

Parametric regression models were developed using seven physicochemical parameters derived from specialty coffee beverages to predict sensory evaluation scores. The results demonstrated that models incorporating the processing method (natural or washed) yielded superior predictions compared to models that ignored this factor, highlighting the importance of considering postharvest processing in sensory quality predictions.

### Data Overview

A summary of the sensory quality ratings provided by the cupping panel is presented in Table 1, categorized by cupping year and processing method. Of the total samples analyzed, 64% were from the year 2019. Sensory scores ranged from 86.9 to 91.1 in 2018 and 87.1 to 92.9 in 2019, with coefficients of variation below 1.36% for both years, indicating consistency in the cupping evaluations. According to the international standard for specialty coffee, these coffees must achieve a cupping score of 80 or higher on a scale of 100 and exhibit no primary defects.^38^ All samples analyzed in this study met or exceeded this threshold, affirming their classification as specialty coffees. This data overview provides a robust foundation for analyzing the performance of the predictive models, ensuring their relevance for assessing high-quality coffees.

**Table 1:** Sensory Evaluation Score for Specialty Coffee Samples with Washed and Natural Process, Obtained by the Panel of Cuppers

	Year			
	2018		2019	
	Washed^a^	Natural^a^	Washed^a^	Natural^a^
# of samples	17	17	38	24
Range	86.9–90.3	87.3–91.1	87.4–92.9	87.1–91.3
Mean ± SD^b^	88.2 ± 0.91	89.0 ± 1.10	89.5 ± 1.22	88.8 ± 0.81

a Process.

b SD = standard deviation.

To assess the effect of the two processing methods (natural and washed) and the year, the Mann–Whitney U test was performed. The results confirmed that both the year and the processing method significantly influence the sensory evaluation scores of the analyzed samples (*p* < 0.05). This finding aligns with previous studies reporting that the processing method affects not only the cup quality but also various sensory attributes and physicochemical parameters.^39,40^

The chemical parameters used in the construction of multivariate models: density (g/mL), Brix, pH, titratable acidity (meq/100g), protein (%), caffeine (mg/L), and 5-CQA (mg/L) are presented in Table 2, categorized by processing method and for all samples combined. The average Brix values were 1.40 for naturally processed samples and 1.44 for washed samples, consistent with previous reports for Panamanian specialty coffees processed similarly.^41^ The pH values also aligned closely with these findings, indicating no substantial deviation in acidity levels between the two methods.

**Table 2:** Physicochemical Characteristics of Specialty Coffee Samples with Washed and Natural Processes

	Density (g/mL)	°Brix	pH	Acidity (meq/100g of extract)	Protein (%)	Caffeine (mg/L)	5-CQA (mg/L)
Natural Process							
Range	0.999–1.002	1.07–1.80	4.80–4.98	1.17–1.56	0.18–0.31	504.60–727.14	912.82–1392.69
Mean ± SD^a^	1.00 ± 0.001	1.40 ± 0.24	4.90 ± 0.04	1.39 ± 0.09	0.23 ± 0.04	633.68 ± 47.06	1154.96 ± 95.75
Washed Process							
Range	0.999–1.003	1.00–1.70	4.79–5.03	1.25–1.97	0.17–0.30	428.01–732.78	755.85–1333.17
Mean ± SD^a^	1.001 ± 0.001	1.44 ± 0.22	4.91 ± 0.05	1.52 ± 0.15	0.23 ± 0.04	605.67 ± 71.66	1070.61 ± 101.31
All samples (Washed + Natural)							
Mean	1.001	1.42	4.90	1.46	0.23	617.64	1106.64
Range	0.999–1.003	1.00–1.80	4.79–5.03	1.17–1.97	0.17–0.31	428.01–732.78	755.85 −1392.69
Mean ± SD^a^	1.001 ± 0.001	1.42 ± 0.23	4.90 ± 0.05	1.46 ± 0.14	0.23 ± 0.04	617.64 ± 63.61	1106.64 ± 107.02

a SD = standard deviation.

The concentration of 5-CQA ranged from 912.82 to 1392.69 mg/L in naturally processed coffees and from 755.85 to 1333.17 mg/L in washed samples. These levels were significantly lower than those reported by Salamanca-Neto,^42^ who identified 5-CQA concentrations ranging from 2361 to 4026 mg/L in specialty coffees. However, the values obtained in this study were within the range reported for coffee arabica samples from Brazil.^43^ Chlorogenic acids (CGAs), particularly 5-CQA, are important contributors to the sensory quality and potential health benefits of coffee.^44^ The variation in CGA levels between the two processing methods reinforces the influence of postharvest techniques on coffee composition and quality. These findings emphasize the need to consider processing methods in studies evaluating sensory and chemical attributes, as they play a critical role in defining the quality characteristics of specialty coffee.

The Mann–Whitney U test was applied to evaluate the effect of the processing method and year on the physicochemical parameters. The results showed a significant influence of the processing method on density, acidity, caffeine, and 5-CQA, while the year had a significant effect on Brix and protein.

### Correlation Analysis of Sensory Score and Physicochemical Parameters

Evaluating coffee beverage quality involves more than measuring the levels of chemical compounds such as caffeine, chlorogenic acids, trigonelline, and aromatic compounds present in the beans. It also requires consideration of how these compounds’ concentrations vary due to the physical and chemical transformations resulting from different production and processing methods.^40,45^

**Figure 1:**
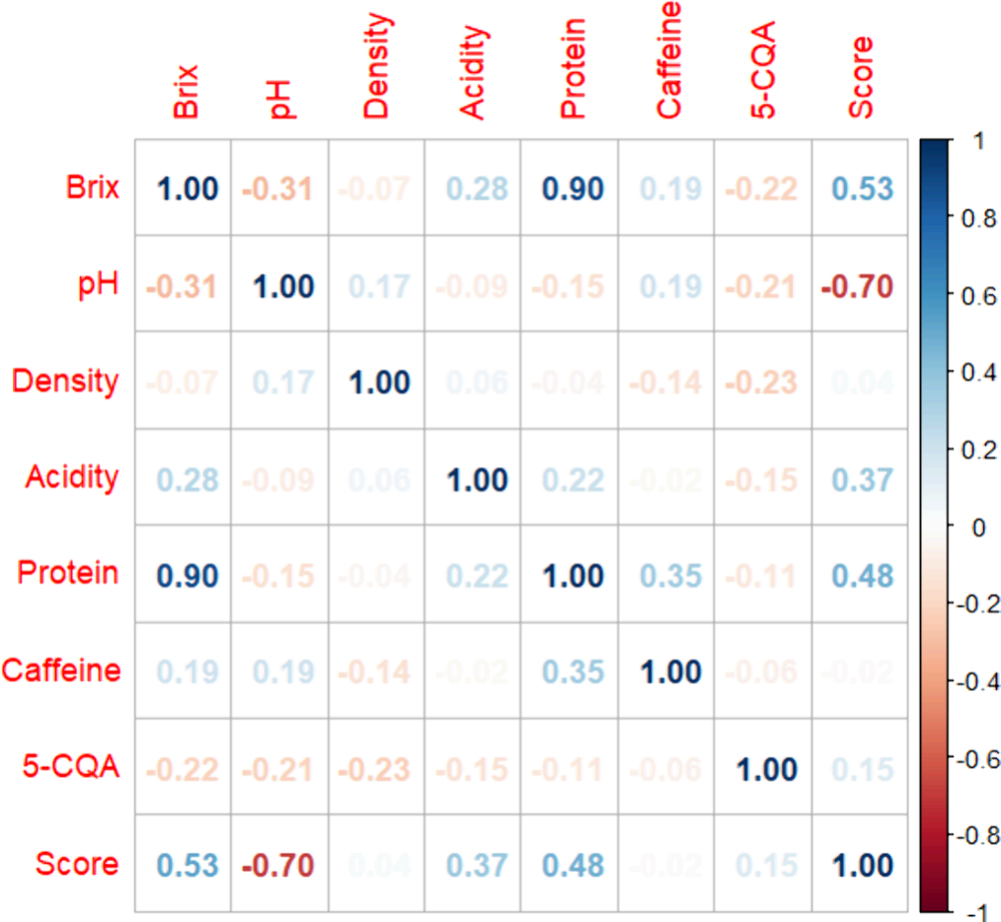
Pearson correlation coefficients (r) of the physicochemical characteristics and sensory quality score of specialty coffee samples.

Figure 1 illustrates the significant correlations between physicochemical parameters and sensory scores as determined by the cupping panel. A positive correlation (**0.3673**) was observed between titratable acidity and sensory scores. This aligns partially with findings by Cortés et al., who reported a high correlation (**0.90**), while Freitas et al. documented a low negative correlation (**−0.01**) between these variables.^46,47^

Soluble solids (Brix) showed positive correlations with titratable acidity (**0.2772**), protein content (**0.8970**), and sensory scores (**0.5274**). Previous studies have noted a weak correlation between soluble solids and sensory evaluations.^47^ Conversely, Brix exhibited negative correlations with pH and 5-CQA, suggesting intricate relationships among these parameters. A negative correlation was identified between pH and sensory scores (**−0.6979**), contrasting with Freitas et al., who reported a positive correlation.^47^ This inverse relationship may highlight variations arising from differences in processing methods or coffee origins. These results underscore the complex interplay between physicochemical characteristics and sensory attributes in coffee. Perceptions of certain attributes, such as acidity or sweetness, can significantly influence the evaluation of others, as suggested in prior research.^47,48^ Understanding these nuanced relationships is essential for enhancing both production techniques and quality assessments of specialty coffee.

### Principal Component Analysis

**Figure 2:**
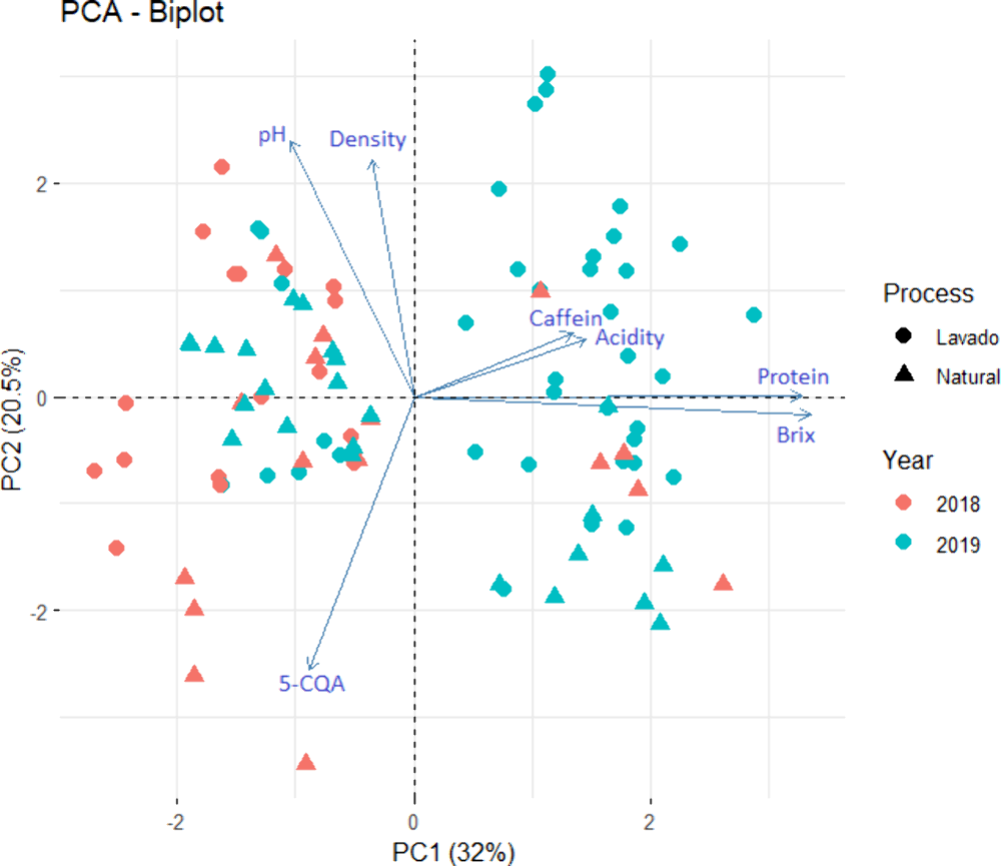
Principal component analysis of physicochemical characteristics of specialty coffee samples identified by year and processing method.

The principal component analysis (PCA) conducted on the sensory scores and physicochemical parameters of the 96 specialty coffee samples revealed that the first principal component (PC1) and the second principal component (PC2) explained 32.0% and 20.5% of the total variability, respectively, accounting for 52.5% of the total variability. This substantial percentage highlights the ability of these two components to represent the underlying structure of the data set. As illustrated in Figure 2, the variables located in the positive direction of the PC1 axis included caffeine, titratable acidity, protein, and Brix. Conversely, density, pH, and 5-CQA concentration were positioned in the negative direction. Among these, protein, and Brix exhibited particularly strong contributions in the positive direction. For PC2, the Brix and 5-CQA concentration were oriented in the negative direction, whereas the other parameters aligned positively. This distinction emphasizes a shift in the relative influence of certain variables in shaping variability within the data set. Figure 2 presents a score chart including all samples, color-coded by year and shape-coded by processing method. Partial differences can be observed both between harvest years (2018 in red and 2019 in blue) and between the two methods (natural represented by triangles and washed by circles). The 2018 samples tend to cluster mostly on the left side, while the 2019 samples are more dispersed on the right side. This analysis provides valuable insights into the multifaceted relationships influencing coffee quality attributes.

### Sensory Evaluation Score Prediction Model and Validation

To develop predictive models for the sensory evaluation scores, 80% of the samples were randomly assigned to the training set. Multiple regression (MLR), principal components regression (PCR), and partial least-squares regression (PLSR) analyses were applied to construct models for a) Specialty coffee samples processed using the washed method, b) Specialty coffee samples processed using the natural method and c) A combined data set including all samples regardless of the processing method.

The residual predictive deviation (RPD) value served as a key criterion for model evaluation. RPD is defined as the ratio between the standard deviation of the prediction set and the root-mean-square error of prediction (RMSEp).^49^ According to established benchmarks, models with an RPD between 2 and 2.5 are considered suitable for quantitative predictions, while values above 2.5 indicate excellent predictive performance.^22^ Models with higher R^2^ values and RPD, alongside lower RMSEp values, are deemed optimal.^49^

### Multiple Linear Regression (MLR) Analysis

To improve the reliability of the predictions, physicochemical parameters were autoscaled as a preprocessing step. Venetian blinds cross-validation, with 10 splits and a blind thickness of 1, was applied to enhance model robustness. The performance metrics, including R^2^ and RMSE for calibration, validation, and prediction, are summarized in Table 3.

**Table 3:** Calibration, Validation, and Prediction Parameters Obtained from the MLR Models, to Predict the Sensory Score of Specialty Coffee with Natural and Washed Process^a^

Multiple linear regression (MLR) models									
	Calibration			Cross-validation		Prediction			
	n	R^2^	RMSE_C_	R^2^_CV_	RMSE_CV_	n	R^2^_P_	RMSEP	RPD
Washed	44	0.7905	0.5532	0.6930	0.6731	11	0.7641	0.6874	2.07
Natural	33	0.9362	0.2297	0.8817	0.3133	8	0.8293	0.4239	2.34
Washed + Natural	77	0.7525	0.5438	0.6954	0.6040	19	0.6140	0.8230	1.51

a *n* = number of samples, R^2^ = coefficient of determination of the calibration set, RMSE_C_ = Calibration root-mean-square error, R^2^_CV_ = cross validation coefficient of determination, RMSE_CV_ = Cross validation root-mean-square error, R^2^_P_ = determination coefficient prediction, RMSE_P_ = root-mean-square prediction error, RPD = residual predictive deviation.

For the combined data set, the MLR model achieved an R^2^ of 0.7525, which was slightly higher than a previous study that reported an adjusted R^2^ of 0.69 using similar chemical parameters, although that study did not provide RMSE or RPD values for comparison.^50^

When considering processing methods individually, the washed coffee model achieved an RPD of 2.07, indicating its suitability for sensory score prediction, the natural coffee model performed slightly better, with an RPD of 2.34, demonstrating a higher level of predictive accuracy and the combined model, which did not differentiate between processing methods, yielded a lower RPD of 1.51, indicating limited predictive utility.

These findings highlight the importance of considering the coffee processing method when developing models to predict sensory scores, as models tailored to specific processes exhibited superior predictive performance. The use of RPD as a robust metric further underscores the reliability of these models for assessing the sensory attributes of specialty coffee.

### Principal Components Regression (PCR) Analysis

**Figure 3:**
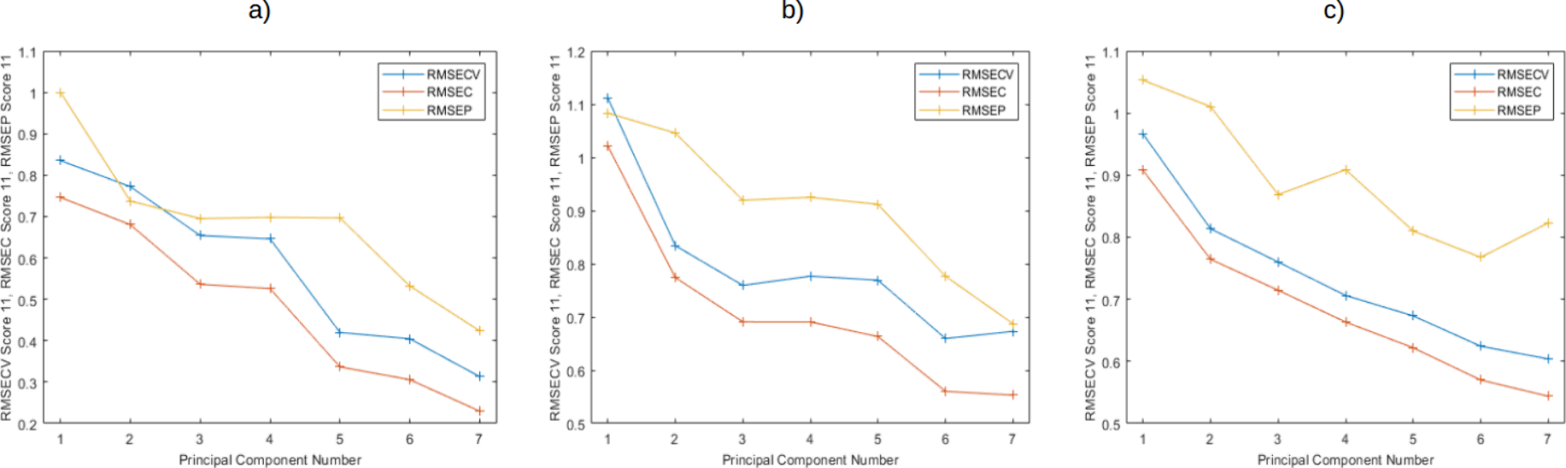
RMSEcv/RMSEc/RMSEp-PCs curves by “Venetian blinds” cross validation protocol of ten data splits for the PCR models for specialty coffee with (a) natural process, (b) washed process, and (c) all samples (washed and natural).

The selection of the optimal number of principal components was determined by comparing the lowest RMSEcv and RMSEp values and the smallest difference between them, which is considered an estimate of the extent of overfitting and a measure of the general applicability of a model. This was done using the cross-validation protocol mentioned earlier (Figure 3).^51,52^

**Figure 4:**
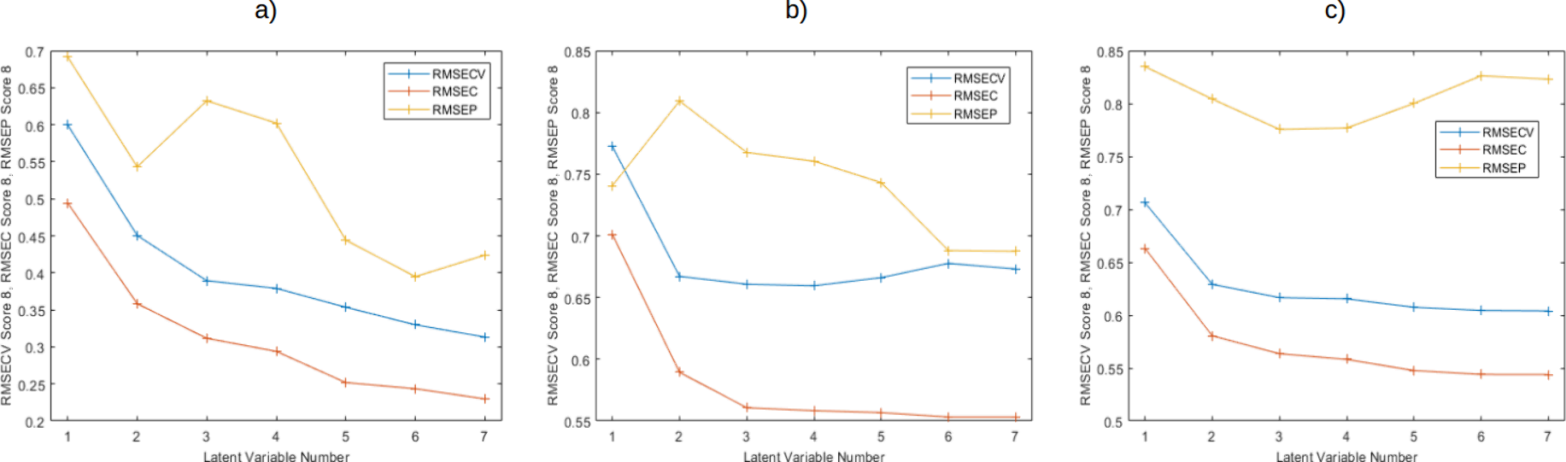
RMSEcv/RMSEc/RMSEp-LVs curves by “Venetian blinds” cross validation protocol of ten data splits for the PLSR models for specialty coffee with (a) natural process, (b) washed process, and (c) all samples (washed and natural).

While the smallest difference between RMSEp and RMSEcv values for the washed process model (Figure 4b) is obtained with 7 PCs, the lowest RMSEcv value is achieved with 6 PCs. Therefore, the latter is chosen as the optimal number of PCs for this data set.

To evaluate the performance of different multivariate methods, principal components regression (PCR) analysis was conducted. The results are summarized in Table 4. Across all models, the PCR approach demonstrated lower R^2^ values for both calibration and prediction compared to the multiple linear regression (MLR) models, indicating reduced explanatory and predictive capabilities. The residual predictive deviation (RPD) values for the three models for natural process coffee, washed process coffee and combined samples (natural and washed) were1.83, 1.86, and 1.62 respectively. These RPD values fall below the threshold of 2.0, indicating that the PCR models are not suitable for quantitative prediction of sensory scores.

**Table 4:** Calibration, Validation, and Prediction Parameters Were Obtained from the PCR Models to Predict the Sensory Score of Specialty Coffee with Natural and Washed Processes^a^

Principal components regression (PCR) model										
		Calibration			Cross-validation		Prediction			
	PC	n	R^2^	RMSE_C_	R^2^_CV_	RMSE_CV_	n	R^2^_P_	RMSE_P_	RPD
Washed	6	44	0.7849	0.5605	0.7037	0.6597	11	0.7173	0.7767	1.83
Natural	6	33	0.8868	0.3059	0.8038	0.4046	8	0.7233	0.5322	1.86
Washed + Natural	6	77	0.7281	0.5700	0.6740	0.6244	19	0.6555	0.7677	1.62

a *n* = number of samples, PC = principal components, R^2^ = coefficient of determination of the calibration set, RMSE_C_ = calibration root-mean-square error, R^2^_CV_ = cross validation coefficient of determination, RMSE_CV_ = root square error cross-validation, R^2^_P_ = coefficient of determination of prediction, RMSE_P_ = root-mean-square error of prediction, RPD = residual predictive deviation.

When considering R^2^, RMSE, and RPD values for both calibration and prediction, it is evident that the PCR models lack the predictive strength necessary for accurately estimating the sensory scores of the specialty coffee samples. This reinforces the need to explore alternative methods, such as PLSR or process-specific models, to improve predictive performance.

### Partial Least-Squares Regression (PLSR) Analysis

The final multivariate statistical technique investigated for predicting the sensory score of specialty coffee produced in Panama was Partial Least Squares Regression (PLSR). Consistent with the previous analytical approaches applied to Multiple Linear Regression (MLR) and Principal Component Regression (PCR) models, an identical preprocessing methodology was employed to develop the PLSR models. The comprehensive results of this analysis are presented in Table 5, revealing coefficient of determination (R^2^) and Root Mean Square Error of Prediction (RMSEP) values remarkably similar to those obtained from the MLR models. Notably, a significant advancement was observed in the Ratio of Performance to Deviation (RPD) for natural coffee samples, which reached an impressive value of 2.51. Referencing the established performance criteria used in this study, this RPD value demonstrates a usable predictive capacity. Specifically, the PLSR model exhibits an outstanding ability to estimate the sensory score of specialty coffee processed using the natural method, based on the predetermined physicochemical parameters. This finding underscores the potential of PLSR as a robust and reliable analytical approach in sensory score prediction for specialty coffee.

**Table 5:** Calibration, Validation, and Prediction Parameters Were Obtained from the PLSR Models to Predict the Sensory Score of Specialty Coffee with Natural and Washed Processes^a^

Partial least-squares regression analysis (PLSR) model										
		Calibration			Cross-validation		Prediction			
	LV	n	R^2^	RMSE_C_	R^2^_CV_	RMSE_CV_	n	R^2^_P_	RMSE_P_	RPD
Washed	6	44	0.7905	0.5532	0.6891	0.6777	11	0.7639	0.6891	2.07
Natural	6	33	0.9283	0.2435	0.8693	0.3299	8	0.8306	0.3948	2.51
Washed + Natural	4	77	0.7392	0.5582	0.6835	0.6154	19	0.6483	0.7769	1.60

a n = number of samples, LV = latent variables, R^2^ = coefficient of determination of the calibration set, RMSE_C_ = calibration root-mean-square error, R^2^_CV_ = cross validation coefficient of determination, RMSE_CV_ = root square error cross-validation, R^2^_P_ = coefficient of determination of prediction, RMSE_P_ = root-mean-square error of prediction, RPD = residual predictive deviation.

An inadequate selection of the optimal number of LVs can lead to an “underfitting” or “overfitting” issue by choosing a number of LVs lower or higher than the optimal one. For the PLS models in this study, the optimal number of LVs was determined using a 10-fold “venetian blinds” cross-validation protocol. The result for the model of samples with a natural process (Figure 4a) with 6 LVs corresponds to the lowest RMSEp and RMSEcv values and the smallest difference between them, which serves as an indicator of potential overfitting and reflects the model’s generalizability.^53,54^ Therefore, 6 LVs were selected as the optimal number of latent variables for this model. The same strategy was used to determine the most suitable LVs for the other two models (Figure 4).

**Figure 5:**
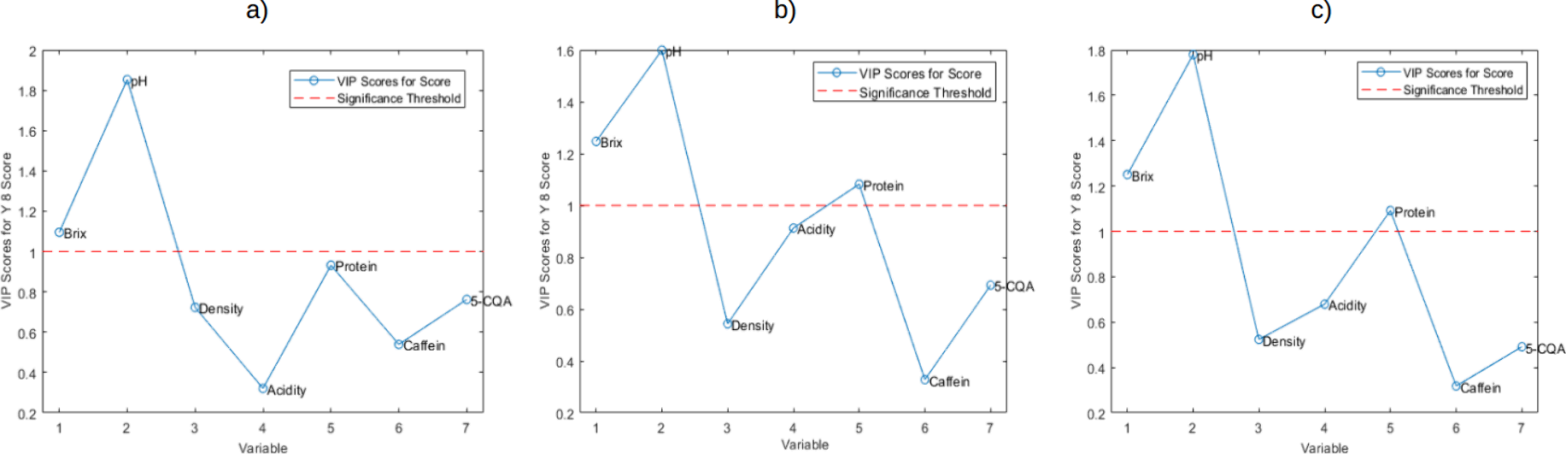
Variable importance in projection (VIP) graph for 7 physicochemical parameters for the PLSR models for specialty coffee with (a) natural process, (b) washed process, and (c) all samples (washed and natural).

Figure 5 presents the VIP plots displaying the physicochemical parameters that contributed the most to the sensory score. The *x*-axis represents each parameter used in the study, while the *y*-axis shows the VIP score, which indicates the importance of each parameter in predicting the sensory score for the PLSR models of specialty coffee with (a) natural process, (b) washed process, and (c) all samples (washed and natural). VIP values greater than 1 (values above the dashed red horizontal line) are considered significantly important for predicting the score. Accordingly, Brix and pH exhibited the highest contribution in all three final PLS models, along with protein in the models for washed process samples (b) and all samples (c).

**Figure 6:**
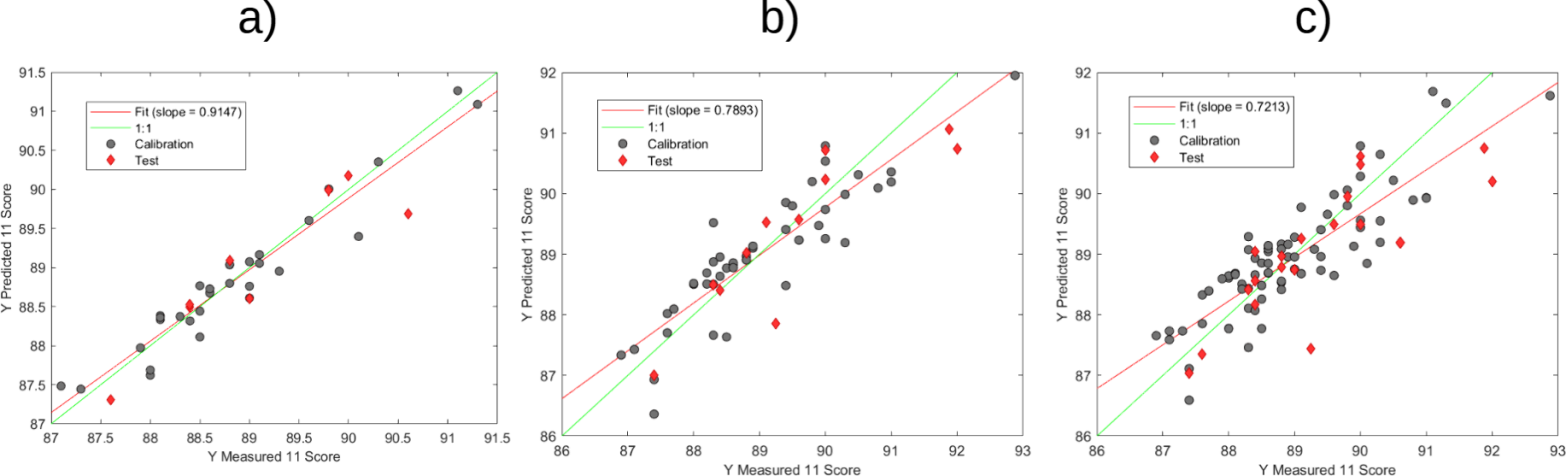
Scatter plots of measured sensory score versus predicted sensory score with PLSR models for specialty coffee with (a) natural process, (b) washed process, and (c) all samples (washed and natural).

The validation of the Partial Least Squares Regression (PLSR) models’ predictive performance was conducted through a comprehensive analytical approach. Initially, a scatter plot (Figure 6) was generated to visually compare the actual sensory score values with their corresponding predicted values, providing an intuitive graphical representation of the model’s predictive accuracy. To rigorously validate the statistical reliability of the PLSR models, a paired Student’s *t* test was performed with a significance level of *p* < 0.05. This statistical method was specifically designed to assess whether systematic differences existed between the actual sensory scores and the model-predicted values for both natural and washed coffee processing methods.

The statistical analysis yielded critical insights into the models’ predictive capabilities. The paired *t* test results revealed no statistically significant differences between the real and predicted sensory evaluation scores (p-value <0.05). This finding is pivotal, as it statistically corroborates the models’ accuracy and reliability across different coffee processing techniques. Consequently, the developed PLSR models for both natural and washed coffee processes demonstrate robust predictive capabilities for specialty coffee sensory evaluation scores. The combination of graphical visualization and rigorous statistical testing provides strong scientific validation of the models’ performance, highlighting their potential utility in sensory score prediction for specialty coffee research and quality assessment.

## Conclusions

The comprehensive analysis revealed nuanced insights into the relationship between physicochemical parameters and specialty coffee sensory evaluation. Specifically, titratable acidity, Brix, and protein content demonstrated positive correlations with sensory scores, while pH exhibited inverse relationships, highlighting the complex biochemical interactions that contribute to coffee quality.

A critical methodological finding emerged regarding chemometric model development: the coffee processing method fundamentally influences predictive accuracy. Models constructed using samples from a single processing technique (either washed or natural) consistently outperformed those incorporating mixed processing methods. This underscores the importance of maintaining methodological homogeneity in sensory score prediction models. Among the multivariate statistical approaches evaluated, Partial Least Squares Regression (PLSR) emerged as the most promising technique for predicting coffee beverage sensory scores. The PLSR models demonstrated superior calibration statistics, particularly when considering the Ratio of Performance to Deviation (RPD) across the investigated physicochemical parameters.

While the current study provides a robust preliminary framework, the research also identified significant opportunities for future investigation. The potential exists to enhance model performance through the strategic incorporation of additional chemical parameters. However, this will require meticulous selection to ensure meaningful contributions to predictive accuracy.

The authors emphasize that this study represents an initial exploration of chemometric techniques for sensory quality prediction in specialty coffee. Future research directions include expanding the range of analyzed chemical parameters, investigating the impact of different *Coffea arabica* varieties, particularly high-quality cultivars like Geisha, refining chemometric modeling techniques to improve predictive precision. Geisha variety’s demonstrated superior quality suggests that varietal characteristics could provide significant insights into sensory score prediction, representing a promising avenue for subsequent research.
